# The Addition of Hypomethylating Agents to Low-Intensity Induction Chemotherapy Does Not Improve Outcomes in Elderly Acute Myeloid Leukemia Patients: A Single-Center Retrospective Study

**DOI:** 10.3390/medicina59010114

**Published:** 2023-01-06

**Authors:** Dan Liu, Xiaoyu Wang, Juan Tong, Li Zhou, Erling Chen, Ziwei Zhou, Lei Xue, Xuhan Zhang, Guangyu Sun, Changcheng Zheng

**Affiliations:** 1 Department of Hematology, Anhui Provincial Hospital, WanNan Medical College, Wenchang Xi Road No. 22, Wuhu 241002, China; 2 Department of Hematology, The First Affiliated Hospital of USTC, Division of Life Sciences and Medicine, University of Science and Technology of China, Lujiang Road No. 17, Hefei 230001, China

**Keywords:** acute myeloid leukemia, elderly patients, low intensity chemotherapy, hypomethylating agents, complete remission, survival

## Abstract

*Background and Objectives:* This study aimed to evaluate whether the addition of hypomethylating agents (HMA) to low-intensity chemotherapy can enhance the clinical efficacy of induction treatment for elderly acute myeloid leukemia (AML) patients who are unsuitable for standard induction therapy. *Materials and Methods:* This study retrospectively analyzed 117 patients over 60 years old who were initially diagnosed with AML and received low-intensity induction treatment in the Department of Hematology in Anhui provincial hospital from January 2015 to December 2020. Twenty-three patients were excluded, and the remaining 94 patients were divided into two groups according to the selection of induction regimens. *Results:* Forty-four patients received HMA combined with low-intensity chemotherapy, and the other 50 patients received only low-intensity induction chemotherapy. Forty-three patients (45.7%) obtained complete remission (CR) after the initial induction treatment. The CR rate in the HMA plus low-intensity chemotherapy group was 34.1% (15/44), and in the single low-intensity chemotherapy group was 56.0% (28/50) (*p* = 0.04). The 30 days cumulative early death rates were 9.1% (95% CI: 3.5–22.4%) in the HMA plus low-intensity chemotherapy group and 6.0% (95% CI: 2.0–17.5%) in the single low-intensity chemotherapy group, respectively (*p* = 0.59), and the one-year cumulative relapse rates were 21.1% (95% Cl: 9.8–41.9%) and 33.3% (95% Cl: 20.3–51.5%), respectively (*p* = 0.80). The one-year overall survival (OS) rates for patients in the HMA plus low-intensity chemotherapy group and the single low-intensity chemotherapy group were 37.3% (95% Cl: 23.1–51.5%) and 55.4% (95% Cl: 40.5–67.9%), respectively (*p* = 0.098), and the one-year event-free survival (EFS) rates were 8.5% (95% Cl: 2.2–20.6%) and 20.6% (95% Cl: 9.1–35.3%), respectively (*p* = 0.058). *Conclusions:* This study showed that the addition of HMA to low-intensity induction chemotherapy does not improve prognosis in elderly AML patients who are unsuitable for standard induction chemotherapy.

## 1. Introduction

Acute myeloid leukemia (AML) is a common hematological malignancy in adults. The median age of patients is 68 years old, and more than half of the patients are over 60 years old when first diagnosed [1]. The traditional “3 + 7” regimen of cytarabine and anthracycline is the first-line induction therapy for AML patients, and the five-years survival rate is 30–35% for patients younger than 60 years old, and for the elderly patients, the five-years survival rate is only 12.5%, and the early mortality rate is 20–50% [2,3]. The toxicity of chemotherapy may outweigh the benefit for the patients who are older, in poor performance, or not suitable for standard-intensity chemotherapy, and the median survival time was about 6–8 weeks when only receiving the supportive treatment [4,5]. Therefore, it is appropriate for clinicians to select low-intensity treatment for elderly patients who are not suitable for standard-intensity induction.

With the development of epigenetic research, hypomethylating agents (HMA) including decitabine (DEC) and azacytidine (AZA) which can mainly reactivate the silenced tumor suppressor gene due to aberrant DNA methylation, are considered to be the most effective regimens for the treatment of myelodysplastic syndrome (MDS) and AML [6,7]. However, most patients would be relapsed because the single-regimen HMA therapy cannot remove all the malignant clones [8]. Therefore, new, and more effective low-intensity induction schemes need to be further explored. Recently, it has been reported that the complete remission (CR) rate is 67% and the survival time has been prolonged when patients received HMA and venetoclax, a Bcl-2 inhibitor, which has become the new hope for patients who cannot tolerate standard-intensity induction [9]. However, for patients with poor economic conditions who cannot use venetoclax or in areas where venetoclax cannot be supplied, low-intensity chemotherapy is still an important treatment option for elderly AML patients.

In this retrospective study, we compared the CR rate, minimal residual disease (MRD) negative rate, overall survival (OS) and event-free survival (EFS) rates between low-intensity chemotherapy and HMA combinations in newly diagnosed elderly AML patients who are unsuitable for standard induction therapy, in order to evaluate whether the addition of HMA to low-intensity induction chemotherapy could improve the prognosis of these patients.

## 2. Materials and Methods

### 2.1. Patients

This study retrospectively analyzed 117 patients over 60 years old who were initially diagnosed with AML and received low-intensity induction treatment in the Department of Hematology in Anhui provincial hospital from January 2015 to December 2020. All patients underwent bone marrow aspiration and were diagnosed according to morphology, immunology, cytogenetics, and molecular biology (MICM). Among the patients studied, we excluded 23 patients who received only single HMA induction therapy and divided the remaining 94 patients into two groups according to the selection of induction regimens. Forty-four patients received HMA combined with low-intensity chemotherapy, and the other 50 patients received only low-intensity induction chemotherapy. We collected patient data from the hospital’s electronic medical record system. The characteristics of the patients in both groups at their first visit are shown in Table 1. The protocol was approved by the Ethics Committee of Anhui Provincial Hospital (approval number: 2022-RE-144) and carried out in accordance with the Declaration of Helsinki.

### 2.2. Treatment

We chose one of the following induction treatments, which were not overlapped and repeated. Fifty patients in the single low-intensity chemotherapy group: 34 patients received low-dose IA regimen (idarubicin, 5–10 mg/m^2^/day, for 3 days; and low-dose cytarabine 10 mg/m^2^/day, for 7 days); 5 patients received low-dose DA regimen (daunorubicin, 20–40 mg/m^2^/day, for 3 days; and low-dose cytarabine 10 mg/m^2^/day, for 7 days); 6 patients received CAG regimen (low-dose cytarabine 10 mg/m^2^, Q12 h, for 14 days, aclarubicin 7 mg/m^2^/day, for 8 days; or 14 mg/m^2^/day for 4 days, G-CSF 5 ug/kg/day for 14 days); 5 patients received HAG regimen (homoharringtonine, 1 mg/m^2^/day, for 7 days, low-dose cytarabine 10 mg/m^2^, Q12 h, for 14 days, G-CSF 5 ug/kg/day for 14 days). Forty-four patients in HMA plus low-intensity chemotherapy group: Among them, 34 patients were treated with DEC (15–20 mg/m^2^/day for 5 days): there were 6 patients of DEC + CAG regimen, 10 patients of DEC+ low-dose IA regimen and 18 patients of DEC + HAG regimen. The other 10 patients were treated with AZA (75 mg/m^2^/day for 7 days), including 7 patients of AZA + low-dose IA and 3 patients of AZA + low-dose cytarabine.

Patients who achieved CR/CRi entered consolidation treatment phase and were treated with sequential low-dose chemotherapy regimens (such as low dose-IA or DA, CAG, HAG, HMA + low-dose cytarabine, HMA +CAG, HMA + HAG, etc.).

### 2.3. Definitions and Statistical Analysis

Early death was defined as death from any cause from the start of diagnosis to the first 30 days. The overall response rate (ORR) included CR, complete remission with incomplete hematologic recovery (CRi) and partial remission (PR). Overall survival (OS) was defined from initial diagnosis to death from any cause or the time until follow-up. Event-free survival (EFS) was defined from initial diagnosis to relapse or death. Cumulative incidence of relapse (CIR) was defined from CR to disease relapse. MRD was examined by analysis of bone marrow fluid using an eight-color flow cytometer, which was identified by leukemia-associated immunophenotype, and MRD levels < 0.01% were considered negative. The continuous variables were compared between the two groups using the Mann-Whitney U test, and categorical variables were compared using the chi-square test or Fisher’s exact test. The probability of OS and EFS is calculated according to the Kaplan Meier curve. The cumulative incidence rate function method was used to estimate the probability of early death and relapse. The statistical analysis was carried out using R statistical software (R statistical calculation basis). Differences with *p* < 0.05 were considered significant.

## 3. Results

### 3.1. Clinical Characteristics

The median age was 70 years (range 60–83) in the whole cohort. The median age of patients in HMA plus low-intensity chemotherapy group was 69 years old (range 60–83), and there were 8 (18.2%) patients over 75 years old. The median age of patients in single low-intensity chemotherapy group was 70 years old (range 60–83), and 7 (14.0%) patients were over 75 years old. According to the available cytogenetic and molecular biological data, risk stratification was carried out according to the 2017 European leukemia (ELN) guidelines [10]. In HMA plus low-intensity chemotherapy group, 35 (79.5%) patients belonged to the favorable- or intermediate-risk group, and 9 (20.5%) patients belonged to the adverse-risk group. In the single low-intensity chemotherapy group, 42 (84.0%) patients belonged to the favorable- or intermediate-risk group, and 8 (16.0%) patients belonged to the adverse-risk group.

### 3.2. Response to Induction Therapy

**Figure 1:**
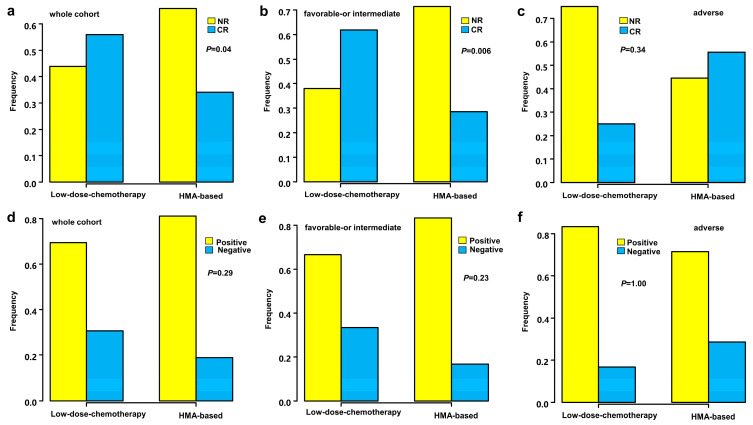
The CR/MRD after the first course of induction chemotherapy in the low dose-chemotherapy group and HMA-based group:(**a**) CR for the whole cohort; (**b**) CR for favorable- or intermediate-risk patients; (**c**) CR for adverse-risk patients; (**d**) MRD for the whole cohort; (**e**) MRD for favorable- or intermediate-risk patients; and (**f**) MRD for adverse-risk patients. Abbreviations: HMA-based, hypomethylating agents combined with low dose chemotherapy; CR, complete remission; MRD, minimal residual disease; NR, non-CR.

Forty-three patients (45.7%) obtained CR after initial induction treatment. The CR rate in the HMA plus low-intensity chemotherapy group was 34.1% (15/44), and in the low-intensity chemotherapy group was 56.0% (28/50) (*p* = 0.04) (Figure 1a). According to the risk stratification of ELN, there were 36 (46.8%) patients with CR in favorable- or intermediate-risk cohort after initial induction, including 28.6% (10/35) in the HMA plus low-intensity chemotherapy group and 62.0% (26/42) in the single low-intensity chemotherapy group (*p* = 0.006) (Figure 1b). In the adverse-risk cohort, there were 7 (41.2%) patients with CR after initial induction, including 55.6% (5/9) in the HMA plus low-intensity chemotherapy group and 25.0% (2/8) in the single low-intensity chemotherapy group (*p* = 0.34) (Figure 1c).

After the initial induction treatment, 73 patients were examined for MRD by flow cytometry: 18.9% (7/37) was MRD-negative in the HMA plus low-intensity chemotherapy group and 30.6% (11/36) was MRD-negative in the low-intensity chemotherapy group (*p* = 0.29) (Figure 1d). In the favorable- or intermediate-risk cohort, 16.7% (5/30) of patients receiving HMA plus low-intensity chemotherapy had MRD-negativity, and 33.3% (10/30) of patients receiving low-intensity chemotherapy had MRD-negativity (*p* = 0.23) (Figure 1e). In the adverse-risk patients, 28.6% (2/7) of patients receiving HMA plus low-intensity chemotherapy had MRD-negativity, and 16.7% (1/6) of patients receiving low-intensity chemotherapy had MRD-negativity (*p* = 1.00) (Figure 1f).

### 3.3. Early Death

**Figure 2:**
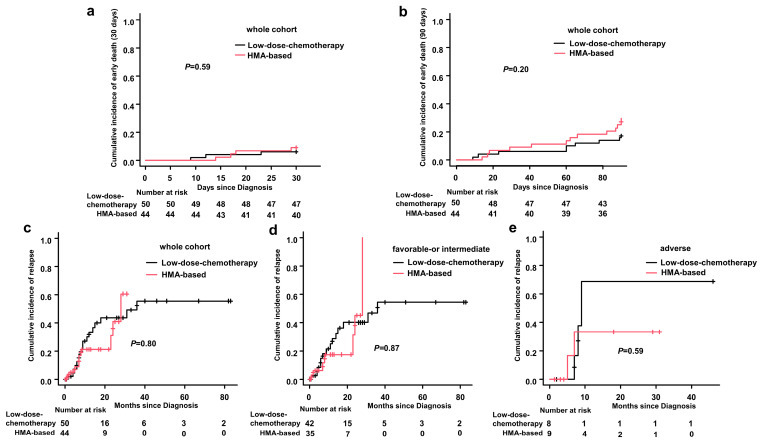
Early death and relapse: (**a**) early death in the first 30 days for the whole cohort; (**b**) early death in the first 90 days for the whole cohort; (**c**) relapse for the whole cohort; (**d**) relapse for favorable- or intermediate-risk patients; and (**e**) relapse for adverse-risk patients. Abbreviations: HMA-based, hypomethylating agents combined with low dose chemotherapy.

During the induction treatment, the incidence of serious infection (pulmonary fungal infection, severe pneumonia, sepsis, etc.) in the HMA plus low-intensity chemotherapy group and the single low-intensity chemotherapy group were 54.5% vs. 52.0%, respectively (*p* = 0.838). Within 30 days after the first diagnosis, seven patients (7.4%) died: four patients (9.1%) in the HMA plus low-intensity chemotherapy group, including two patients with cerebral hemorrhage and two patients with severe infection and multiple organ failure. Three patients (6.0%) died in the single low-intensity chemotherapy group, including two patients with cerebral hemorrhage and one patient with severe infection. The 30 days cumulative early death rates were 9.1% (95% CI: 3.5–22.4%) in the HMA plus low-intensity chemotherapy group and 6.0% (95% CI: 2.0–17.5%) in the single low-intensity chemotherapy group, respectively (*p* = 0.59) (Figure 2a).

A total of 22 patients (23.4%) died within 90 days after the first diagnosis, including 13 patients (29.5%) in the HMA plus low-intensity chemotherapy group and nine patients (18.0%) died in the single low-intensity chemotherapy group. The 90 days cumulative death rates were 29.5% (95% CI: 18.4–45.4%) in the HMA plus low-intensity chemotherapy group and 18.0% (95% CI: 9.8–31.7%) in the single low-intensity chemotherapy group (*p* = 0.20) (Figure 2b).

### 3.4. Disease Relapse

During consolidation and follow-up, the one-year cumulative relapse rates in the HMA plus low-intensity chemotherapy group and the single low-intensity chemotherapy group were 21.1%(95% Cl: 9.8–41.9%) and 33.3%(95% Cl: 20.3–51.5%), respectively (*p* = 0.80) (Figure 2c). Among the groups with favorable- or intermediate-risk patients, the one-year cumulative relapse rates in the HMA plus low-intensity chemotherapy group and the single low-intensity chemotherapy group were17.5%(95% Cl: 6.6–41.6%) and 28.6%(95% Cl: 15.9–48.1%), respectively (*p* = 0.87) (Figure 2d). In the adverse-risk patients, the one-year cumulative relapse rates in the HMA plus low-intensity chemotherapy group and the single low-intensity chemotherapy groups were 33.3% (95% Cl: 9.6–80.5%) and 68.8% (95% Cl: 26.6–98.7%), respectively (*p* = 0.59) (Figure 2e).

### 3.5. OS and EFS

**Figure 3:**
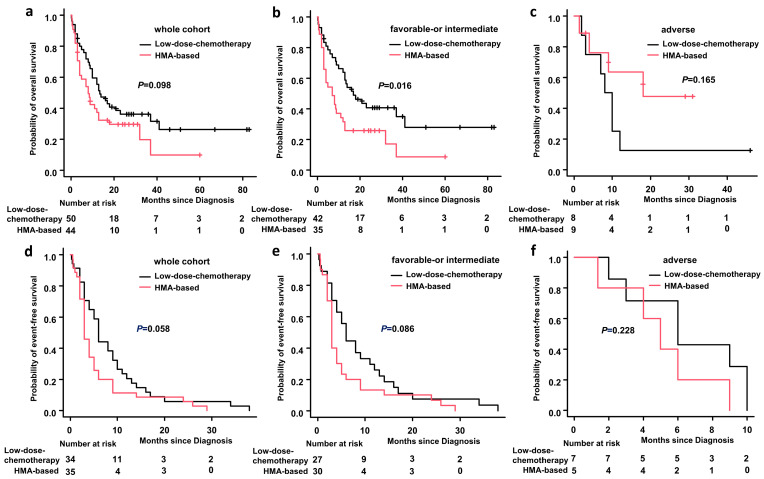
Overall survival and event-free survival: (**a**) overall survival (OS) for the whole cohort; (**b**) OS for favorable- or intermediate-risk patients; (**c**) OS for adverse-risk patients; (**d**) event-free survival (EFS) for the whole cohort; (**e**) EFS for favorable- or intermediate-risk patients; and (**f**) EFS for adverse -risk patients. Abbreviations: HMA-based, hypomethylating agents combined with low dose chemotherapy.

All patients were followed up to 31 March 2022 through our electronic medical record system or outpatient or telephone contact, and the information was reliable. At the end of follow-up, the median OS of patients in the HMA plus low-intensity chemotherapy group was eight months (range, 0.3–67). The median OS of patients in the single low-intensity chemotherapy group was 13.5 months (range, 2–83). The one-year OS rates for patients in the HMA plus low-intensity chemotherapy group and the single low-intensity chemotherapy group were 37.3% (95% Cl: 23.1%–51.5%) and 55.4% (95% Cl: 40.5–67.9%), respectively (*p* = 0.098) (Figure 3a). For favorable- or intermediate-risk patients, the one-year OS rate was significantly higher in the single low-intensity chemotherapy group than in the HMA plus low-intensity chemotherapy group, 63.7% (95% Cl: 47.2–76.3%) and 31.4% (95% Cl: 17.1–46.8%), respectively (*p* = 0.016) (Figure 3b). For adverse-risk patients, the one-year OS rates for patients in the HMA plus low-intensity chemotherapy group and the single low-intensity chemotherapy group were 47.6% (95% Cl: 12.3%–76.9%) and 12.5% (95% Cl: 0.7–42.3%), respectively (*p* = 0.165) (Figure 3c). The one-year EFS rates for patients in the HMA plus low-intensity chemotherapy group and the single low-intensity chemotherapy group were 8.5% (95% Cl: 2.2–20.6%) and 20.6% (95% Cl: 9.1–35.3%), respectively (*p* = 0.058) (Figure 3d). For favorable- or intermediate-risk patients, the 1-year EFS rates for patients in the HMA plus low-intensity chemotherapy group and the single low-intensity chemotherapy group were 10.0% (95% Cl: 2.5–23.6%) and 18.5% (95% Cl: 6.7–34.8%) (*p* = 0.086) (Figure 3e). For adverse-risk patients, the one-year EFS rates for patients in the HMA plus low-intensity chemotherapy group and the single low-intensity chemotherapy group were 20.0% (95% Cl: 0.84–58.2%) and 28.6% (95% Cl: 4.1–61.2%) (*p* = 0.228) (Figure 3f).

## 4. Discussion

At present, there is no literature report on whether the addition of HMA on the basis of low-intensity chemotherapy can improve the poor prognosis of elderly AML. It is unclear whether HMA can enhance the clinical efficacy of low-intensity chemotherapy for elderly AML patients, including raising the CR rate, reducing the relapse rate and prolonging the survival.

In most clinical circumstances, monotherapy with HMA (azacitidine or decitabine) or low-dose cytarabine was recommended for older adults with AML not suitable for intensive antileukemic therapy. Until now, there are only two clinical studies have proved that combination treatment is effective in elderly AML. A multicenter phase II clinical trial [11] showed that for AML patients who were not eligible for intensive chemotherapy (n = 116), the use of low-dose cytarabine combined with glasdegib prolonged survival OS to 8.3 months in the combination group and only 4.3 months in the group with low-dose cytarabine alone, (*p* = 0.0002), with manageable adverse effects. Dinardo et al. [9] proved that venetoclax combined with HMA was well tolerated in elderly AML, with an increased overall response rate (ORR) of 67%. However, this study shows that the addition of HMA to low-intensity chemotherapy did not improve the prognosis of elderly AML. There were no significant differences in the one-year cumulative relapse rate and the one-year OS rate in the combined HMA group compared to the single low-intensity chemotherapy group (*p* = 0.802, 0.098). Especially in the patients with favorable- or intermediate-risk, the use of the single low-intensity chemotherapy regimen resulted in a significantly higher CR rate than in the combined HMA group (62.0% vs. 28.6%) (*p* = 0.006) and a significantly higher one-year OS rate (63.7% vs. 31.4%) (*p* = 0.016). Although the combination of HMA in this study did not increase the incidence of severe infection events, it has been reported [12] that the incidence of agranulocytosis with fever (infection) increased significantly (42% vs. 19%) when venetoclax was combined with AZA. Another phase III multicenter randomized open trial [13] showed that comparing low-intensity cytarabine with DEC, the risk of severe agranulocytosis (24% and 15%) and infection (21% and 15%) was significantly increased in the DEC group.

Poor cytogenetic characteristics and secondary AML in elderly patients are the main causes of poor prognosis and affect the long-term survival [14]. Although this study shows that the addition of HMA to the initial induction cannot improve the survival of elderly AML, in certain clinical situations, such as treatment of patients with complex karyotype, previous MDS history, TP53 mutation, HMA-based chemotherapy might be favored, because patients with adverse biology are not likely to respond to induction with low-intensity chemotherapy only, such as low-dose cytarabine. Xing-Nong et al. [15] conducted a clinical trial to investigate the effectiveness of the sequential combination of DEC followed by low-dose chemotherapy in high-risk myeloid neoplasms and found that epigenetic priming with DEC had an increased anti-leukemia effect. This study also supports the additional use of HMA to low-intensity chemotherapy in patients with adverse-risk patients; although the statistical difference is not significant, the combined HMA treatment group has a higher CR rate (55.6% vs. 25.0%), a longer one-year OS rate (47.6% vs. 12.5%) and a lower one-year cumulative relapse rate (33.3% vs. 68.8%).

## 5. Conclusions

In conclusion, this study showed that the addition of HMA to low-intensity induction chemotherapy did not significantly improve the outcome in elderly AML patients who are unsuitable for standard induction chemotherapy. In particular, patients in the favorable- or intermediate-risk groups are more likely to achieve CR and prolong OS using single low-intensity chemotherapy. However, this is a retrospective clinical study with a limited number of patients in each group; and the regimens in low-intensity chemotherapy were heterogeneous which includes low-dose IA, DA, CAG and HAG, etc. A large number of patients with unified low-intensity chemotherapy regimens should be enrolled in a prospective study in the future.

## Figures and Tables

**Table 1:** Patient demographics and baseline characteristics.

	HMA-Based n = 44	Low-Dose-Chemotherapy n = 50	*p* Values
Age (years), median (range)	69 (60–83)	70 (60–83)	0.539
Age ≥ 75 years, n (%)	8 (18.2)	7(14.0)	0.767
Sex: M/F, n (%)	21/23 (47.7/52.3)	30/20 (60.0/40.0)	0.300
WBC at first diagnosis (×10^9^/L), median (range)	6.8 (0.52–172.05)	5.5 (0.83–139.2)	0.264
PLT at first diagnosis (×10^9^/L), median (range)	56.5 (6–532)	52.5 (4–285)	0.557
LDH level at first diagnosis, median (range)	302 (153–11,372)	346 (127–889)	0.771
CD56 positive expression, n (%)	6 (13.6)	11 (22.0)	0.421
ECOG-PS, n (%) 0–1 ≥2	19 (43.2) 25 (56.8)	16 (32.0) 34 (68.0)	0.291
Comorbidities, n (%) Hypertension Diabetes Heart disease Cerebral infarction Hepatitis Arrhythmia Tumor Rheumatism Fracture Gout lumbar disc protrusion Eye diseases Hypothyroidism Mental illness Pulmonary tuberculosis Syphilis Chronic obstructive pulmonary disease None	10 (22.7) 5 (11.4) 0 (0.0) 3 (6.9) 2 (4.5) 0 (0.0) 2 (4.5) 1 (2.3) 1 (2.3) 1 (2.3) 0 (0.0) 1 (2.3) 1 (2.3) 0 (0.0) 0 (0.0) 1 (2.3) 1 (2.3) 15 (34.1)	15 (30.0) 5 (10.0) 1 (2.0) 0 (0.0) 2 (4.0) 2 (4.0) 1 (2.0) 0 (0.0) 2 (4.0) 0 (0.0) 2 (4.0) 1 (2.0) 0 (0.0) 1 (2.0) 1 (2.0) 0 (0.0) 2 (4.0) 15 (30.0)	0.645
Two or more comorbidities, n (%)	9 (20.5)	6 (12.0)	0.398
Molecular biology, n (%) NPM1 mutation FLT3 mutation FLT3 mutation MPM1 mutation Biallelic mutated CEBPA CEBPA mutation CEBPB mutation CBFB-MYH11 mutation AML1-ETO mutation MLL arrangements DNMT3A mutation TET-2 mutation CSF3R mutation Negative detection No data	6 (13.6) 2 (4.5) 1 (2.3) 1 (2.3) 0 (0.0) 1 (2.3) 1 (2.3) 1 (2.3) 1 (2.3) 1 (2.3) 1 (2.3) 0 (0.0) 19 (43.2) 9 (20.5)	5 (10.0) 3 (6.0) 3 (6.0) 3 (6.0) 1 (2.0) 0 (0.0) 0 (0.0) 2 (4.0) 0 (0.0) 0 (0.0) 0 (0.0) 1 (2.0) 29 (58.0) 3 (6.0)	0.178
ELN risk assessment, n (%) Favorable-risk Intermediate-risk High-risk	10 (22.7) 25 (56.8) 9 (20.5)	8 (16.0) 34 (68.0) 8 (16.0)	0.558
Response after one course of induction, n (%) CR CRi PR ORR	15 (34.1) 3 (6.8) 6 (13.6) 24 (54.5)	28 (56.0) 4 (8.0) 4 (8.0) 36 (72.0)	0.040 1.000 0.579 0.090
Severe infection at first diagnosis, n (%)	24 (54.5)	26 (52.0)	0.838

Abbreviations: HMA-based, hypomethylating agents combined with low dose chemotherapy; WBC, white blood cell; PLT, platelet; LDH, lactate dehydrogenase; ECOG-PS, Eastern Cooperative Oncology Group Performance Status; ELN, European Leukemia Net; CR, complete remission; CRi, complete remission with incomplete hematologic recovery; PR, partial remission; ORR, overall response rate.

## Data Availability

The datasets used and/or analyzed in this study are available from the corresponding authors upon reasonable request.
